# Mouse intraductal modeling of primary ductal carcinoma *in situ*

**DOI:** 10.1016/j.xpro.2023.102526

**Published:** 2023-08-30

**Authors:** Stefan J. Hutten, Fariba Behbod, Colinda L.G.J. Scheele, Jos Jonkers

**Affiliations:** 1 Division of Molecular Pathology, The Netherlands Cancer Institute, 1066CX Amsterdam, The Netherlands; 2 Oncode Institute, Amsterdam, The Netherlands; 3 Department of Pathology and Laboratory Medicine, The University of Kansas Medical Center, Kansas City, KS 66103, USA; 4 Center for Cancer Biology, VIB, Department of Oncology, KU Leuven, 3000 Leuven, Belgium

**Keywords:** Cell Biology, Cancer, Microscopy, Model Organisms

## Abstract

Mouse intraductal modeling enables efficient *in vivo* propagation of pre-invasive breast cancer lesions and provides a suitable micro-environment for creating patient-derived tumor xenograft models of estrogen-receptor-positive breast cancer. Here, we present a protocol for mouse intraductal modeling of primary ductal carcinoma *in situ* (DCIS). We describe steps for processing primary DCIS tissues and performing intraductal injections. We then detail procedures for processing intraductal lesions for 3D whole-mount imaging or serial transplantation using magnetic bead sorting.

For complete details on the use and execution of this protocol, please refer to Hutten et al. (2023).^1^

## Highlights

• Generation of mouse intraductal (MIND) models of ductal carcinoma *in situ* (DCIS)
• Protocol for 3D whole-mount imaging of mammary glands
• Sequential transplantation of DCIS-MIND models with magnetic bead sorting

## Abstract (editor highlights)

Publisher’s note: Undertaking any experimental protocol requires adherence to local institutional guidelines for laboratory safety and ethics.

## Graphical abstract

**Figure:**
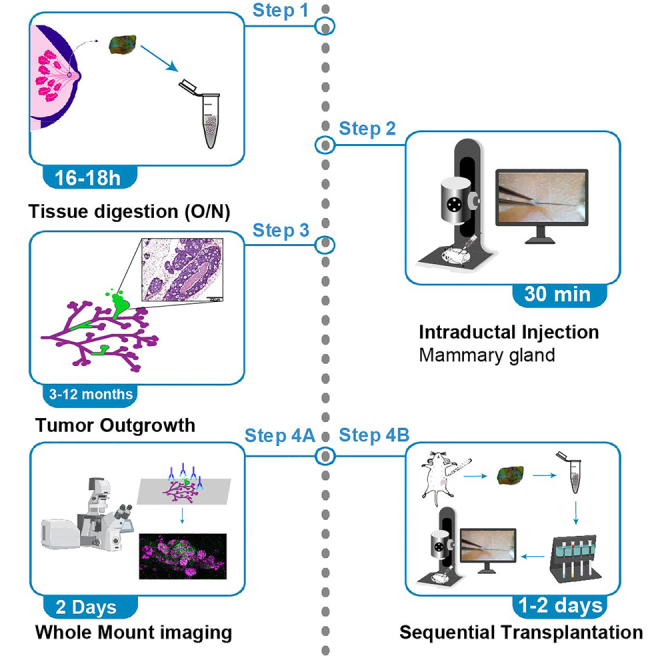

## Before you begin

*In vivo* modeling of human breast cancer is primarily done by generating patient-derived xenografts (PDX) models via implantation of tumor fragments in the mammary fat pad of immune deficient mice. This works well for aggressive breast cancers such as triple-negative breast cancer, but is not well suited for hormone receptor-positive luminal breast cancer and early stages of breast cancer such as ductal carcinoma *in situ* (DCIS).^2^ Here we describe a protocol for the generation of DCIS PDX models by mouse mammary intraductal (MIND) injection of primary DCIS cells, 3D whole mount imaging of DCIS-MIND lesions, and serial transplantation of engrafted DCIS-MIND models.^3^^,^^4^ The protocol below describes the specific steps for using primary human DCIS material. However, we have also used this protocol with primary human invasive breast cancer tissue and cell lines.

### Institutional permissions

Primary DCIS samples are obtained from patients undergoing surgery. The samples should be acquired with informed consent from the subjects and the study should be approved by the institutional review board (IRB). All animal experiments described here were approved by the Animal Welfare Committee of The Netherlands Cancer Institute (NKI) in accordance with national guidelines. Animals were maintained in the animal facility of the NKI, housed in individually ventilated cages (IVC) under specific pathogen-free (SPF) conditions, and received food and water ad libitum.

### Mouse housing

**Timing: 7 days**

***Note:*** We use six- to eight-week-old female NOD.Cg-Prkdc scid Il2rg tm1Wjl /SzJ (NSG) mice (Jackson Laboratory, Strain# 005557; RRID: IMSR_JAX:005557). Mice should be acclimatized in the animal facility for at least one week before intraductal injection.

**CRITICAL:** In the case of Estrogen Receptor (ER) positive samples, provide animals with 17β-estradiol (4 μg/mL) in the drinking water 7 days prior to intraductal injection and keep the animals on estradiol-supplemented drinking water for the whole duration of the experiment.

### Preparation of equipment and materials for intraductal injection

**Timing: 1 day**

Ensure you have all the equipment necessary for intraductal injections (tissues, razor blades, 70% Ethanol, PBS, 15 mL Falcon tube, 20 μL pipet and tips, 1.5 mL Eppendorf tubes, anesthesia induction box, waste box, Evans blue (2 mg/mL), syringes, instruments (microscope (EVO CAM II with TV screen), tweezers, Hamilton syringe) and a flow cabinet with isoflurane.

## Key resources table

**Table:** 

REAGENT or RESOURCE	SOURCE	IDENTIFIER
**Antibodies**		

Biotin mouse anti-mouse H-2K (clone SF1-1.1); 1:100	BD Pharmingen	Cat# 553564; RRID: AB_394922
Biotin mouse anti-mouse I-A (clone AMS-32.1); 1:100	BD Pharmingen	Cat# 553546; RRID: AB_394913
Anti-Biotin MicroBeads	Miltenyi Biotec	Cat# 130-090-485
Anti-human CD326 (EpCAM) eFluor 660 (clone 1B7)	eBioscience	Cat# 50-9326-42; RRID: AB_10598658
Ku80 monoclonal antibody (clone C48E7); 1:100	Cell Signaling Technology	Cat# 2180S
Alpha-smooth muscle actin monoclonal antibody (clone 1A4); 1;1000	eBioscience	Cat# 14-9760-82; RRID: AB_2572996
Donkey anti-rabbit IgG (H + L) highly cross-adsorbed secondary antibody, Alexa Fluor 568; 1:400	Invitrogen	Cat# A10042; RRID: AB_2534017
Goat anti-mouse IgG2a cross-adsorbed secondary antibody, Alexa Fluor 647; 1:400	Invitrogen	Cat# A-21241; RRID: AB_2535810
DAPI	Thermo Fisher	Cat# D1306

**Cell culture reagents**		

Advanced DMEM/F12	Gibco	Cat# 12634-010
HEPES buffer solution (1 M)	Gibco	Cat# 15630-056
GlutaMAX (100×)	Gibco	Cat# 35050-061
Penicillin-Streptomycin (5,000 U/mL)	Gibco	Cat# 15070-063
0.05% Trypsin-EDTA	Gibco	Cat# 25300-054
0.5 M EDTA solution	Lonza	Cat# BMA51201
Bovine serum albumin	Sigma-Aldrich	Cat# A8022
Normal goat serum	Thermo Fisher	Cat# 50197Z

**Chemicals, peptides, and recombinant proteins**		

Hyaluronidase	Sigma-Aldrich	Cat# H3884; CAS: 37326-33-3
Collagenase type IV	Thermo Fischer	Cat# 17104019
Collagenase A from *Clostridium histolyticum*	Sigma-Aldrich	Cat# COLLA-RO; CAS: 9001-12-1
Deoxyribonuclease I from bovine pancreas	Sigma-Aldrich	Cat# DN25; CAS: 9003-98-9
17β-estradiol	Sigma-Aldrich	Cat# E2758; CAS: 50-28-2
FcR blocking reagent, human	Miltenyi Biotec	Cat# 130-059-901
Evans blue	Sigma	Cat# E2129; CAS: 314-13-6
Trypan blue	Sigma	Cat# T8154; CAS: 72-57-1
Tween 20	Sigma-Aldrich	Cat# P1379
HBSS	Thermo Fisher	Cat# 14025092
4% Paraformaldehyde in 1× PBS	Santa Cruz	Cat# sc-281692
Triton X-100	Sigma-Aldrich	Cat# X100-1L
L-lysine	VWR	Cat# TS30334; CAS: 56-87-1
Sodium metaperiodate (NAIO_4_)	Millipore	Cat# 106597; CAS: 7790-28-5
NaH_2_PO_4_	Millipore	Cat# 106342; CAS: 13472-35-0
Na_2_HPO_4_	Millipore	Cat# 106586; CAS: 7558-79-4
NH_4_Cl	Sigma-Aldrich	Cat# A9434; CAS: 12125-02-9

**Experimental models: Organisms/strains**		

NOD.Cg-Prkdcscid Il2rgtm1Wjl/SzJ (NSG); 6–8-week-old females	The Jackson Laboratory	Strain# 005557; RRID: IMSR_JAX:005557

**Other**		

LD columns	Miltenyi Biotec	Cat# 130-042-901
70 μm Cell strainer	Corning	Cat# 352350
Glass syringe (50 μL)	Hamilton	Cat# 7637-01
34 Gauge needle (point style 4)	Hamilton	Cat# 207434
Dumont #5 tweezers	Fisher Scientific	Cat# 50-241-57
Stainless double-edge razor blades	SuperMax	N/A
Pipette (P20)	Rainin	Cat# 17008650
Scalpels	Thermo Fisher	Cat# 11738353
Mr. Frosty Freezing Container	Thermo Fisher	Cat# 5100-0001
LUNA-II automated cell counter	Logos Biosystems	N/A
LUNA cell counting slides	Logos Biosystems	Cat# L12001
6 cm Petri dish	Greiner	Cat# 628160
EVO CAM II	Vision Engineering	N/A
QuadroMACS separator	Miltenyi Biotec	Cat# 130-090-976
MACS MultiStand	Miltenyi Biotec	Cat# 130-042-303
Hot plate A4	Labotect	Cat# 16481

## Materials and equipment

**Table:** Advanced DMEM/F12+++ (ADDF+++)

Reagent	Final concentration	Amount
Advanced DMEM/F12 (1×)	1×	485 mL
Penicillin-Streptomycin (5,000 U/mL)	50 units/mL	5 mL
HEPES (1 M)	10 μM	5 mL
GlutaMAX (100×)	1×	5 mL
**Total**	**N/A**	**500 mL**

**CRITICAL:** ADDF+++ can be stored at 4°C for a maximum of 6 months

**Table:** Digestion Solution (per 100 mg)

Reagent	Final concentration	Amount
Collagenase Type IV	152.5 units/mL	5 mg
Hyaluronidase	72 units/mL	0.24 mg
BSA	0.2%	200 mg
ADDF+++	1×	10 mL
**Total**	**N/A**	**10 mL**

**CRITICAL:** Digestion solution is prepared freshly immediately before usage

**Table:** FACS buffer

Reagent	Final concentration	Amount
EDTA solution (0.5 M)	5 mM	2.5 mL
BSA	0.5%	2.5 g
PBS	1×	497.5 mL
**Total**	**N/A**	**500 mL**

**CRITICAL:** FACS buffer can be stored at 4°C for a maximum of 6 months

**Table:** PLP fixative

Reagent	Final concentration	Amount
4% paraformaldehyde in 1× PBS	4%	2.5 mL
NaIO_4_	0.002 g/mL	0.0212 g
L-Lysine in P-buffer (0.2 M)	0.075 M	3.75 mL
P-buffer	N/A	3.75 mL
**Total**	**N/A**	**10 mL**

**CRITICAL:** PLP fixative is prepared freshly immediately before usage

**Table:** Mild-digestion solution

Reagent	Final concentration	Amount
Collagenase A	0.375 U/mL	25 mg
Hyaluronidase (10000 U/mL stock solution)	100 U/mL	10 μL
PBS	1×	10 mL
**Total**	**N/A**	**10 mL**

**CRITICAL:** mild-digestion solution is prepared freshly immediately before usage

**Table:** Blocking buffer

Reagent	Final concentration	Amount
BSA	1%	0.1 g
Triton X-100 (10% stock solution)	0.8%	0.8 mL
Normal goat serum	5%	0.5 mL
PBS	1×	8.7 mL
Total	**N/A**	**10 mL**

**CRITICAL:** blocking buffer can be stored at −20°C for a maximum of 6 months

• DNAse solution: add 9 μL of DNAse (10 U/μL) in 4 mL ADDF+++.

**CRITICAL:** DNAse solution is prepared freshly immediately before usage

• BSA solution: PBS with 2.5% of BSA.

**CRITICAL:** BSA solution can be stored at 4°C for a maximum of 6 months

• Evans Blue: 20 mg of Evans Blue powder in 10 mL of H_2_O.

**CRITICAL:** Evans Blue can be stored at 20°C for a maximum of 6 months

• P-buffer: mix 81 mL of 0.2 M Na_2_HPO_4_ (28.39 g/L demineralized H_2_O, store at 20°C, sterile) with 19 mL of 0.2 M NaH_2_PO_4_ (24 g/L demi H_2_O, store at 20°C, sterile) and add 100 mL of demineralized H_2_O. Adjust pH to 7.4 if necessary.

**CRITICAL:** P-buffer can be stored at 4°C for a maximum of 6 months

• L-lysine in P-buffer: 5.848 g in 200 mL P-buffer. Adjust pH to 7.4 if necessary.

**CRITICAL:** L-lysine in P-buffer can be stored at 4°C for a maximum of 6 months

## Step-by-step method details

### Dissociation of primary DCIS tissue

**Timing: 16–18 h**

**Timing: 5–10 min (for steps 1 and 2)**

**Timing: Overnight ∼16 h (for step 3)**

**Timing: 45–60 min (for steps 4–12)**

In this step primary tissue is mechanically and enzymatically dissociated to a single cell solution suitable for intraductal injection.

1. Dissect resected sample with scalpels in a 6 cm petri dish in a few mL of Digestion solution.

**CRITICAL:** Take the potential presence of human pathogens into account and adhere to the corresponding biosafety protocols.

**CRITICAL:** To preserve tissue viability of the primary tissue, it should be processed as quickly as possible after surgical resection (1–5 h). Keep tissue at 4°C in the meantime.

***Note:*** As a general rule of thumb the tissue pieces should be small enough to be taken up by a 25 mL pipette, but try to cut the tissue pieces as small as possible.

2. Transfer tumor pieces to a 50 mL Falcon tube and add 10 mL of digestion solution per 100 mg of tumor tissue.
3. Incubate in shaker at 100 rpm at 37°C for 16 h overnight.

***Note:*** Continuous movement of the tumor pieces in the digestion solution improves digestion and the final number of cells obtained.

4. Transfer to 15 mL falcon tubes and spin at 1500 × *g* for 10 min.
5. Aspirate supernatant, add 1 mL of pre-warmed Trypsin-EDTA and gently pipet up and down for 1 min at 20°C .
6. Add 9 mL of ADDF+++ and spin at 1500 × *g* for 10 min.
7. Aspirate supernatant and add 9 μL of DNAse solution.
8. Vortex for 3–5 min, add 6 mL ADDF+++ and spin at 1500 × *g* for 10 min.
9. Aspirate supernatant and resuspend pellet in 10 mL ADDF+++ (**Coat pipette with 2.5% BSA solution by pipetting up and down twice**).

***Note:*** Coating of material with BSA minimizes loss of primary cells, which is specifically critical when dealing with small amounts of tissue.

10. Filter solution through a 70 μm cell strainer (**2.5% BSA solution coated**) into a 50 mL falcon tube (**2.5% BSA solution coated**), transfer into a 15 mL falcon tube (**2.5% BSA solution coated** ) and spin at 1500 × *g* for 10 min.
11. Aspirate supernatant and suspend cells in 100 μL of PBS (If a large pellet is present 1 mL of PBS can be used).

***Note:*** keep the cells on ice from this point onwards.

12. Count cells:
  a. Take 10 μL of cell suspension and add 10 μL of trypan blue.
  b. Pipet 10 μL of this mix on a cell counting chamber and count cells with cell counter.
  c. Calculate the volume of cells needed to obtain 25,000 cells in 18 μL, which will be injected in each mammary gland.

***Note:*** The single cell suspension will also contain immune- and stromal cells, which are included in the 25,000 cells.

### Intraductal injections of the mammary gland

**Timing: 20–25 min**

**Timing: 10 min for setup**

**Timing: 10–15 min per mouse**

This section describes the setup and steps for intraductal injections of the mammary gland. For intraductal injection the mouse needs to be immobilized and anesthetized. The nipple of the mouse should also be clearly visible, which is done by carefully shaving the area surrounding the nipple.

**Figure 1:**
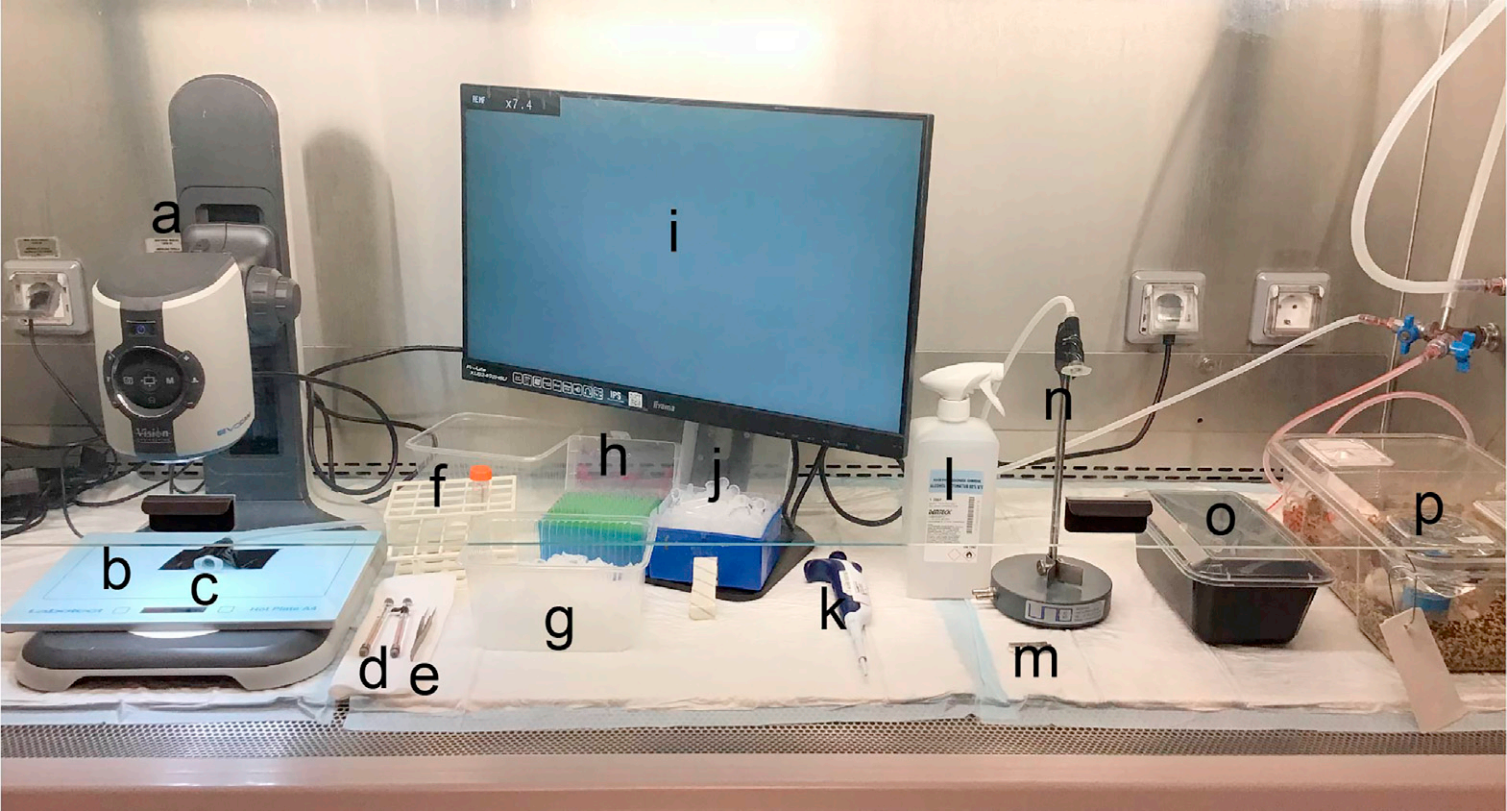
Intraductal injection setup (A) camera. (B) heating pad. (C) 5 mL syringe connected to isoflurane tube. (D) Hamilton syringe. (E) Dumont tweezer. (F) 15 mL Falcon tube with PBS. (G) Ice bucket with samples and stock of Evans Blue. (H) pipet tips. (I) screen. (J) Eppendorf tubes. (K) 20 μL pipet. (L) 70% ethanol. (M) razor blade. (N) 5 mL syringe connected to isoflurane on a standard for shaving. (O) anesthesia induction box. (P) Cage with mice.
14. Anesthetize the mouse with 2.5% isoflurane and 2 L per minute air flow until it falls asleep.
15. Carefully shave around the nipples.
16. Put the mouse in the isoflurane/5 mL syringe under the camera on a heating pad.
17. Prepare droplets of 20 μL (2 μL Evans blue + 18 μL tumor cells) in the lid of an Eppendorf tube and fill the syringe.

***Note:*** First clean the syringe with PBS. Also clean with PBS in between samples.

13. Prepare the setup in a laminar flow hood as shown in Figure 1.
  a. Cover the bottom of the cabinet with 3 sterile diapers.
  b. Install the camera, screen and small heating pad.
  c. Wipe everything that will be in contact with the mouse or you during surgery with disinfectant (e.g., VirkonS) before introduction into the biohazard cabinet or introduce them sterile: tissues, razor blades, 70% Ethanol, PBS, 15 mL Falcon tube, P20 pipet and tips, 1.5 mL Eppendorf tubes, anesthesia induction box, waste box, Evans blue (2 mg/mL), samples on ice, syringes, instruments (tweezers, Hamilton syringe) on a tissue right of the surgical area under the camera.
  d. Turn on heating pad (37°C).
    **CRITICAL:** It is crucial to use the heating pad on 37°C to keep the mouse on the right body temperature.
  e. Connect an anesthesia induction box / 5 mL syringe to the isoflurane station.
    ***Note:*** 5 mL Syringes without plunger can be used as a head cone for the anesthesia for shaving and the injection.

**CRITICAL:** While filling the syringe try to avoid air bubbles, as these can clog the mammary duct, resulting in failure of the injection.

**Figure 2:**
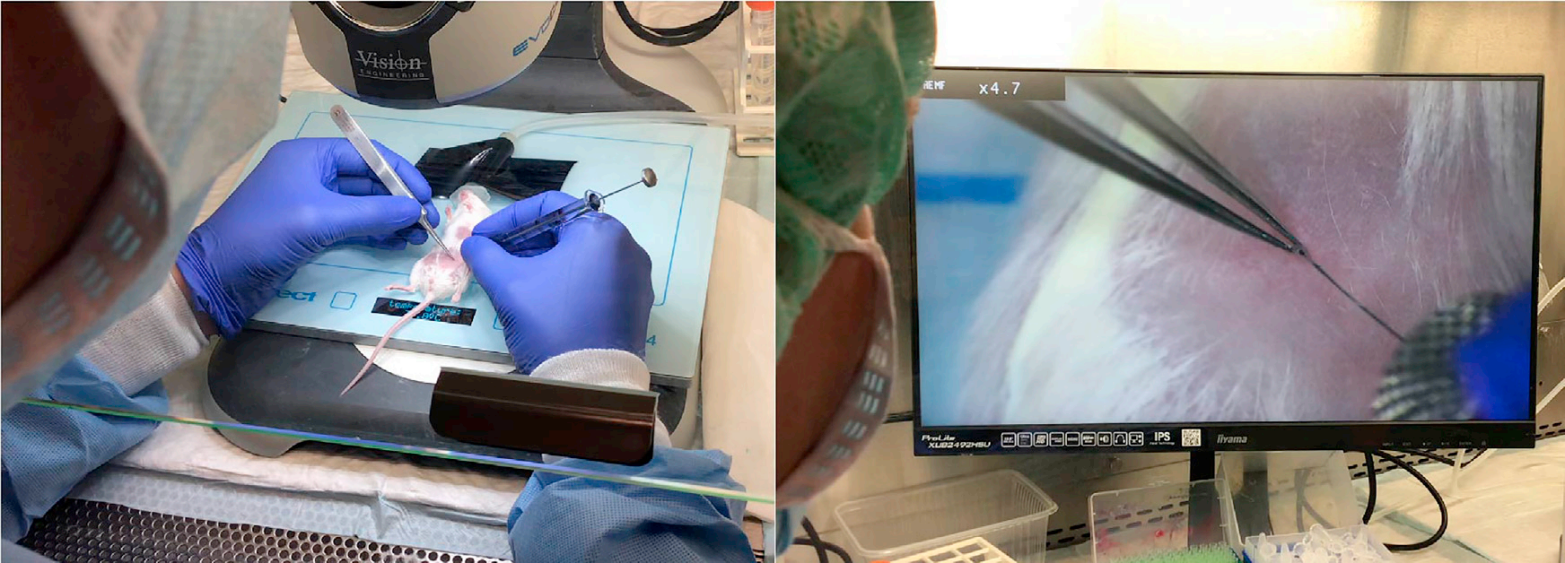
Impression of intraductal injection Also see Methods video S1.

18. Remove the cap of dead skin on the nipple with tweezers (not all nipples have this).
19. Hold the nipple between the tweezers and inject the 20 μL cell suspension in the nipple (see Figure 2 and Methods video S1).

**Figure 3:**
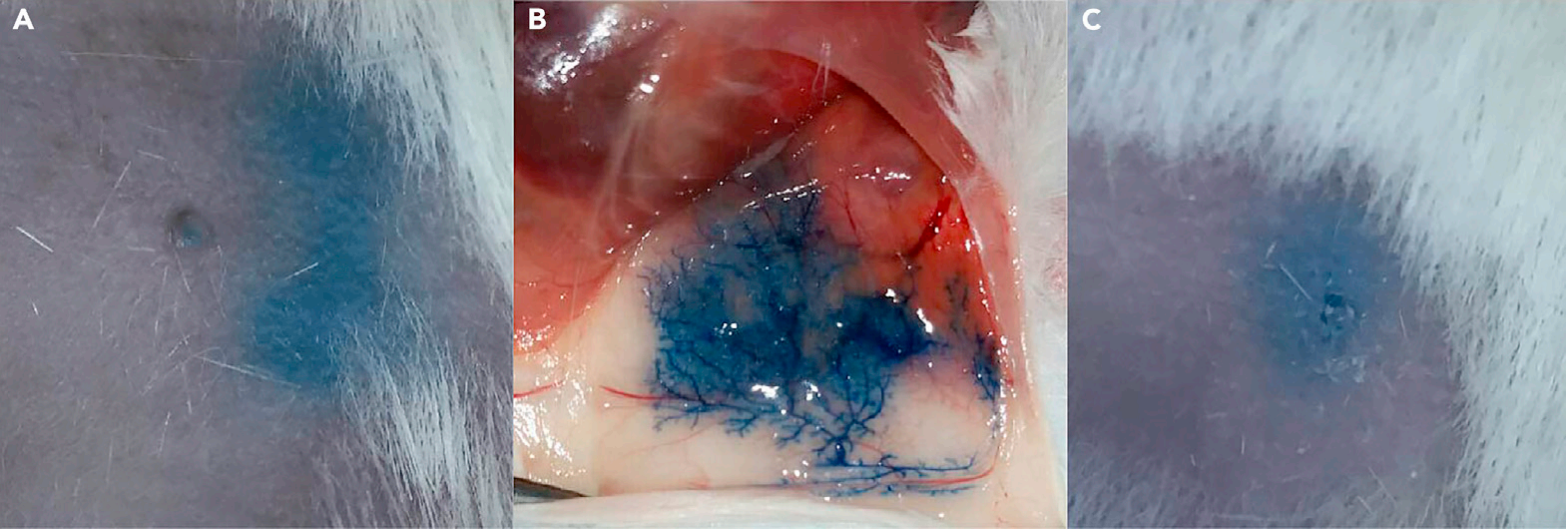
Success intraductal injection (A) Successful intraductal injection. (B) Successful intraductal injection as seen under the skin. (C) Unsuccessful intraductal injection.

**CRITICAL:** It is not necessary to cut the tip of the nipple as described in other protocols.

**CRITICAL:** Check whether the injection was successful if the solution disperses in the mammary gland. A blue bulge directly at the place of injection indicates a failed injection (Figure 3).

***Note:*** Depending on the experiment it is possible to inject multiple mammary glands. The preferred mammary glands are the 3^rd^ and 4^th^ glands on both sides.

***Note:*** The cell suspension should be gradually injected to avoid backflow of the solution.

20. Monitor outgrowth. For primary DCIS samples it takes 6–12 months on average for DCIS lesions to form, which can then be processed further.

Methods video S1. Video of intraductal injection, related to step 19

### 3D whole mount imaging

**Timing: 2 days**

This section describes the protocol to produce 3D whole mounts of mammary glands to image the entire ductal tree and outgrown lesions. This technique can provide information on characteristics such as extent of growth, invasive potential, 3D morphology, growth location etc.

21. Sacrifice the animal and carefully dissect out the injected mammary glands.

***Note:*** Continue with step 36 if you want to use the mammary glands for serial transplantation.

22. Put the mammary gland in a 12- or 24-well plate.

***Note:*** If the tissue already contains endogenous expression of fluorophores, keep it protected from light and cover the plate with aluminum foil.

23. Perform a mild digestion for 20–30 min at 37°C on a shaker (100 rpm) in 2 mL of mild digestion mix.

***Note:*** This step is meant to improve antibody penetration and should not alter the structure of the mammary gland, nor degrade antigen on the cell surface. If any signs of alterations in the tissue structure are observed, decrease the incubation time with mild digestion mix to 10–15 min.

24. Wash the gland three times for 5 min in 2 mL HBSS at 20°C on a shaker (100 rpm).
25. Perform a 2-h fixation in 2 mL PLP at 20°C.
26. Wash the mammary glands twice in 2 mL NH_4_Cl (0.5 M) for 10 min.
27. Wash the gland three times for 10 min in 2 mL PBS at 20°C on a shaker.
28. Add 2 mL blocking buffer for 3 h at 20°C on a shaker.
29. Incubate with selected primary antibodies diluted in 2 mL blocking buffer overnight (±16 h) at 20°C on a shaker.

***Note:*** To image primary human lesions and the myoepithelial cell layer as in Hutten et al.*,*^1^ the following primary antibodies were used: Ku80 Monoclonal Antibody (Clone C48E7, 1:100) for the human epithelial cells and Alpha-Smooth Muscle Actin Monoclonal Antibody (Clone 1A4, 1:1000) for the myoepithelial cells.

30. Wash 3 times for 10 min in 2 mL PBS with 0.2% Tween at 20°C on a shaker.
31. Incubate with secondary antibodies in 2 mL blocking buffer for at least 5 h, protected from light at 20°C on a shaker.

***Note:*** The secondary antibodies used with Ku80 and Alpha-Smooth Muscle Actin were donkey, anti-rabbit Alexa 568 (Invitrogen, A10042. 1:400) and goat anti-mouse IgG2a Alexa 647 (Invitrogen, A21241, 1:400) respectively.

32. Wash for 30 min in 2 mL PBS with 0.2% Tween and DAPI (1 μg/mL) at 20°C on a shaker.
33. Wash 3 times for 20 min in 2 mL PBS with 0.2% Tween at 20°C on a shaker.
34. Mount the mammary gland between 2 coverslips using a whole-gland mounting device.

***Note:*** For a detailed description of the whole-gland mounting device, see Hannezo & Scheele.^5^

**Figure 4:**
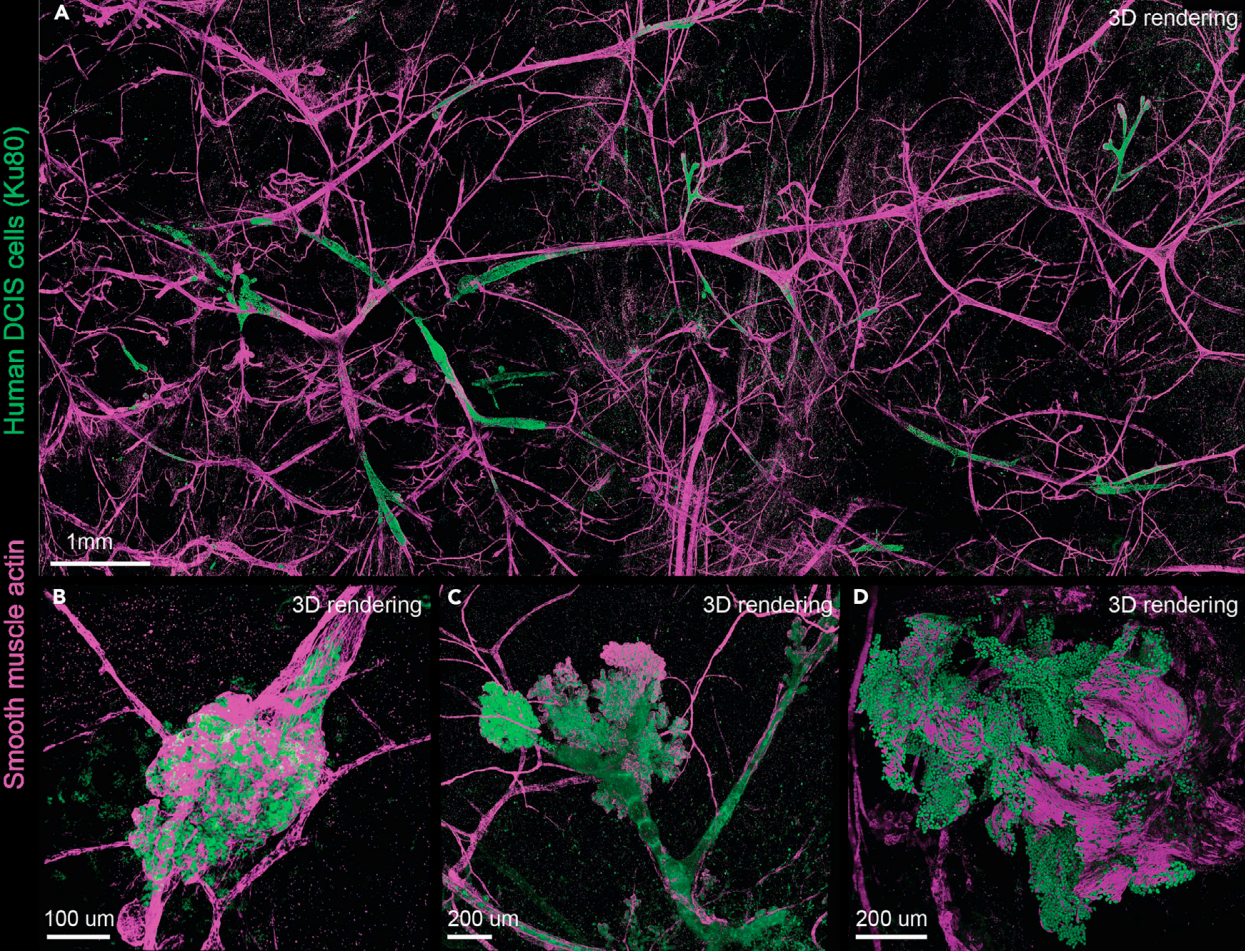
Whole mount images of DCIS-MIND models (A) Overview image showing DCIS lesions spreading in the ductal structures, without disturbing the ductal architecture (replacement growth). (B) Example of a DCIS lesion showing expansive growth. (C) Example of a DCIS lesion showing hyperbranching. (D) Example of a lesion showing invasive growth.

35. Image the tissue using a confocal microscope (see Figure 4).

### Serial transplantation of MIND models

**Timing: ∼20 h**

**Timing: 3 h (for steps 37–58 after overnight digestion)**

This section describes the protocol for processing the outgrowth of the primary tissue in the previous section for serial transplantation. As MIND models often only show intraductal growth it is necessary to separate the human tumor cells from the mouse cells before reinjection. Here we describe a negative selection for unlabeled human cells using magnetic bead sorting. For tumors larger than 500 mm^3^, the following steps are not necessary and only steps 1 to 11 need to be repeated for serial transplantation.

36. Sacrifice the animal and carefully dissect out the injected mammary glands.
37. Repeat step 1 to 11, only suspend cells in 300 μL of cold PBS with 0.5% BSA at step 11.
38. Add 100 μL of human FcR Blocking Reagent for up to 10^8^ total cells (20 μL of FcR per 60 μL buffer/ 10^7^ cells).
39. Label cells with biotinylated antibodies (Mouse Anti-MHC Class I and II) at a dilution of 1:100 (for 400 μL PBS with 0.5% BSA and FcR add 4 μL antibodies). Incubate for 20 min on ice.
40. Wash cells by adding 5–10 mL of PBS with 0.5% BSA per 10^7^ cells and centrifuge at 1500 × *g* for 10 min. Aspirate supernatant completely then repeat wash.
41. Resuspend pellet in 300 μL PBS with 0.5% BSA and add 100 μL of Anti biotin MicroBeads UltraPure (20 μL of beads per 60 μL buffer).
42. Mix well and incubate for 20 min in the refrigerator (2–8°C).
43. Wash cells by adding 5–10 mL of PBS with 0.5% BSA per 10^7^ cells and centrifuge at 1500 × *g* for 10 min.
44. Resuspend up to 10^8^ cells in 500 μL of PBS with 0.5% BSA.

***Optional:*** Save 10 μL for pre-sort flow cytometry.

45. Place LD column in the magnetic field of a suitable MACS Separator.
46. Prepare LD column by rinsing with 2 mL of PBS with 0.5% BSA.

***Note:*** LD Columns are “flow stop” and do not run dry.

47. Discard effluent and change collection tube. The LD Column is now ready for magnetic separation.

***Note:*** Use column immediately after filling to avoid formation of air bubbles caused by warming up. Do not store columns after filling.

48. Apply cell suspension from step 44 onto the column. Collect flow-through containing unlabeled human tumor cells.
49. Wash column with 2 mL of PBS with 0.5% BSA. Collect unlabeled human tumor cells that pass through in the same collection tube as the previous step.

**CRITICAL:** Only start performing the washing step when the column reservoir is empty.

50. Remove column from the separator and place it on a new collection tube.
51. Pipette 2.5 mL of PBS with 0.5% BSA onto the column. Immediately flush out the magnetically labeled cells by firmly pushing the plunger into the column.

***Note:*** These cells can be used as an unstained control for flow cytometry analysis.

52. Spin down the unlabeled cells from step 48 and 49 and resuspend in 110 μL PBS.
  a. Use 10 μL for counting as described previously in step 12.
  b. Use another 10 μL for flow cytometry analysis.
  c. Store the rest of the cell suspension at 4°C for intraductal injection.

### Flow cytometry analysis to detect human cells

This section describes the flow cytometry analysis to determine the number of human tumor cells available for serial transplantation.

53. Label cells from step 52b with 1 μL of human specific EPCAM-e660 antibody (1:100), add 90 μL of cold FACS buffer and incubate for 20 min on ice covered with aluminum foil.

***Optional:*** Also perform this and the following steps with the 10 μL saved for pre-sort flow cytometry from step 44.

54. Wash cells by adding 5 mL of cold FACS buffer and centrifuge at 1500 × *g* for 5 min at 4°C.
55. Aspirate supernatant and suspend cells in 500 μL of cold FACS buffer and add 5 μL of DAPI (1:100).
56. Perform flow cytometry analysis on these cells, the unstained cells from step 51 (control) and the pre-sort sample (optional).
57. Calculate the number of human tumor cells in the sample by combining the percentage of EPCAM positive cells and the number of cells counted in step 52a.
58. Calculate the volume of cells needed to obtain 25,000 human cells in 18 μL, which will be used for serial transplantation by intraductal injection in each mammary gland.

***Note:*** At this point it is also possible to freeze down single-cell solution for later use. Only consider this when you have more than 500,000 cells.

## Expected outcomes

**Figure 5:**
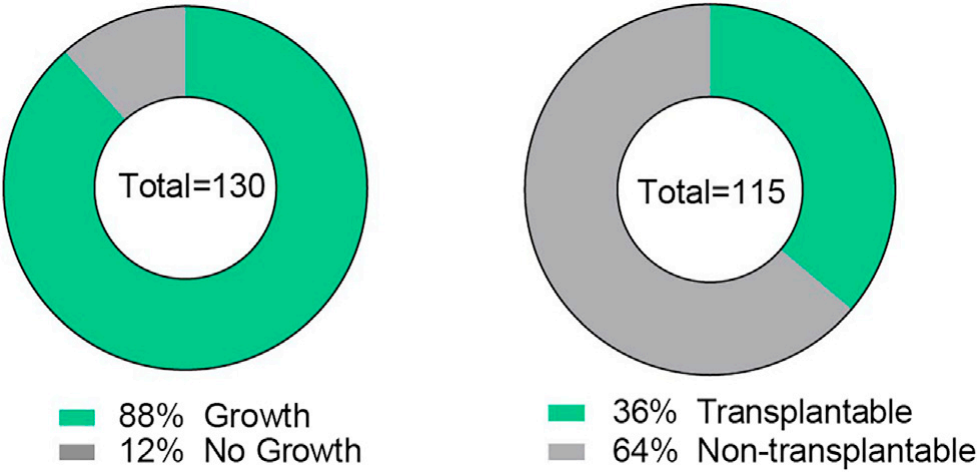
Engraftment rate of DCIS-MIND transplantation (A) percentage of successful engraftment of primary DCIS cells using MIND. (B) percentage of samples with successful serial transplantation.

Using this method, we were able to engraft primary DCIS lesions with a success rate of 88% at first passage, while a smaller percentage of 36% was successfully passaged serially as most DCIS lesions grow slow by nature (Figure 5). DCIS samples were injected into 1 to 9 mice depending on the sample size, with highly varying growth speeds between samples, ranging from palpable tumors within 6 months to microscopic lesions after 12 months. For more details see Hutten et al.^1^

**Figure 6:**
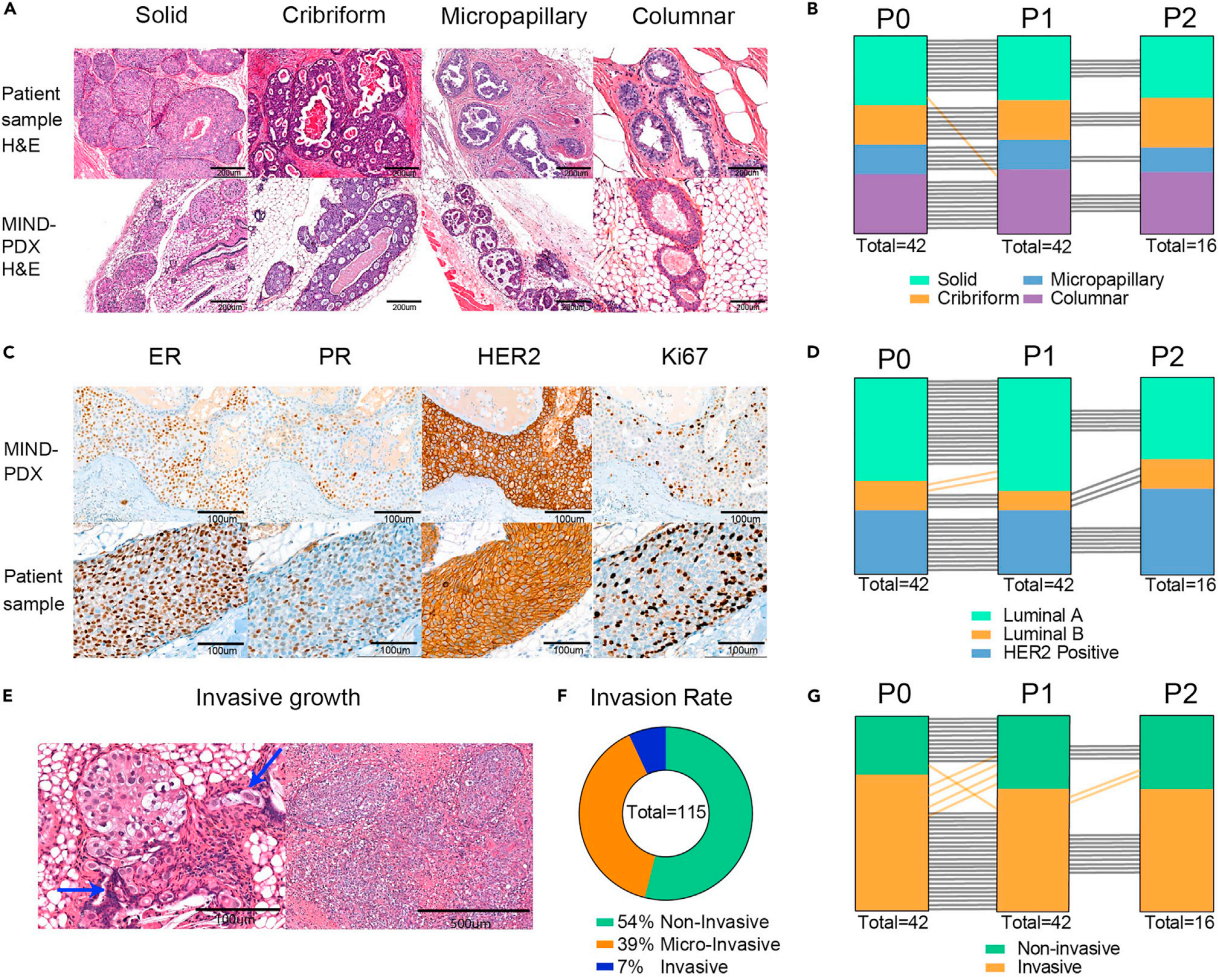
DCIS MIND models retain features of the original tumors (A) H&E staining of DCIS samples from patients and corresponding DCIS-MIND models, showing the different matching growth patterns. (B) Maintenance of growth pattern over multiple passages. Black lines indicate concordance between passages, whereas orange lines indicate a discordance between passages. (C) Examples of immunohistochemistry for estrogen receptor (ER),progesterone receptor (PR), HER2 and Ki67 expression in MIND-DCIS lesions (Top row) versus matched primary DCIS lesions (Bottom row). (D) Maintenance of molecular subtype over multiple passages. Black lines indicate concordance between passages, whereas orange lines indicate a discordance between passages. E) H&E staining of DCIS-MIND models showing micro-invasion (left) or invasive growth (right). Blue arrows indicate micro-invasive cells. (F) Pie chart of the percentage of MIND-DCIS lesions with non-invasive growth, micro-invasion or invasive progression. (G) Maintenance of invasive or non-invasive growth over multiple passages. Black lines indicate concordance between passages, whereas orange lines indicate a discordance between passages.

The DCIS-MIND models retain the features of the original tumor (e.g., growth pattern, ER, progesterone receptor and HER2 expression, as well as genomic aberrations) over multiple passages. As most DCIS-MIND models do not show palpable lesions, end-point analysis such as determining growth pattern and invasive progression is performed by histology and 3D whole mount imaging (Figure 6).

**Figure 7:**
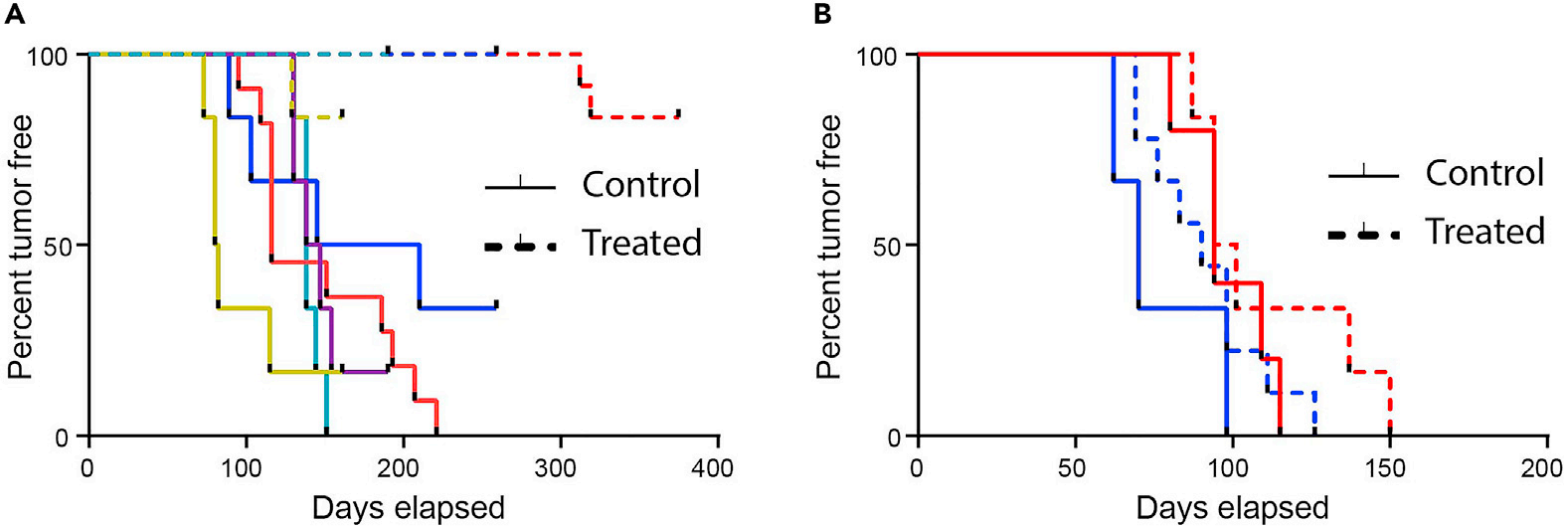
Intervention study (A) Example of Kaplan-Meier curves of trastuzumab-sensitive HER2+ DCIS-MIND models (n = 7). (B) Example of Kaplan-Meier curves of trastuzumab-resistant HER2+ DCIS-MIND models (n = 2).

Transplantable models can also be used to perform drug intervention studies, such as treatment of HER2-positive DCIS models with trastuzumab (Figure 7). Tumor free survival can be quantified for DCIS models with palpable tumors, otherwise it is possible to calculate extent of tumor growth at the end of treatment using H&E or whole mount analysis.

## Limitations

In order to create PDX-MIND models, immunodeficient mice are required, which results in the lack of immune cells and human stroma. As the tumor microenvironment has been shown to be important in many studies, it can be crucial to mimic the human stroma and immune system. Efforts to create immune deficient mouse models with humane immune cells have been made, but these do not yet fully recapitulate the human immune system.^6^

Compared to fat pad or subcutaneous injections, this technique is more difficult to master and requires multiple training sessions which can be time-consuming.

Additionally, as the volume of the ductal tree is limited, the maximum injection volume is around 30 μL, thus limiting the number of cells that can be injected to around 25,000 per mammary gland.

## Troubleshooting

### Problem 1

Insufficient time to perform intraductal injections the day after receiving the primary sample (step 2).

### Potential solution

There are two possible solutions. The first possibility is to only digest the primary tissue for 2–3 h instead of the overnight digestion. You can now fit the whole protocol in 1 day. The second option is to store the primary tissue. Here we recommend mechanically dissociating the primary tissue in small pieces and storing the tissue in 90% FBS with 10% DMSO by first cooling with −1°C /min to −80°C and then transferring to −150°C for long term storage. This option reduces the viability, but also provides the opportunity to receive samples from different locations.

### Problem 2

Slow growth kinetics and lesions that are not palpable (Step 20).

### Potential solution

We recommend injection of 25,000 cells per mammary gland, but it is possible to inject a higher number of cells which might result in faster tumor formation. Another option is to first transduce the cells with luciferase to follow the growth kinetics of the lesions by non-invasive bioluminescence imaging as has been shown by Hutten et al.^1^ and Fiche et al.^7^

### Problem 3

The mouse strain you prefer to use has small nipples, resulting in difficulties to perform intraductal injection (Step 19).

### Potential solution

Provide estradiol in the drinking water of the mice a few days prior to intraductal injections. The estradiol results in enlarged nipples, which are easier to inject.

## Resource availability

### Lead contact

Further information and requests for resources and reagents should be directed to and will be fulfilled by the lead contact, Jos Jonkers, j.jonkers@nki.nl.

### Materials availability

This study did not generate new unique reagents.

## Data Availability

This study did not generate a dataset or code.
